# Association of Adherence to Weight Telemonitoring With Health Care Use and Death: A Secondary Analysis of a Randomized Clinical Trial

**DOI:** 10.1001/jamanetworkopen.2020.10174

**Published:** 2020-07-10

**Authors:** Sarah C. Haynes, Daniel J. Tancredi, Kathleen Tong, Jeffrey S. Hoch, Michael K. Ong, Theodore G. Ganiats, Lorraine S. Evangelista, Jeanne T. Black, Andrew Auerbach, Patrick S. Romano

**Affiliations:** 1 Center for Health and Technology, Department of Pediatrics, University of California, Davis, Sacramento; 2 Center for Healthcare Policy and Research, Department of Pediatrics, University of California, Davis, Sacramento; 3 Adventist Heart and Vascular Institute, St Helena, California; 4 Center for Healthcare Policy and Research, Department of Public Health Sciences, University of California, Davis, Sacramento; 5 Division of General Internal Medicine and Health Services Research, University of California, Los Angeles; 6 Veterans Affairs Greater Los Angeles Healthcare System, Los Angeles, California; 7 Department of Family Medicine and Public Health, University of California, San Diego School of Medicine, La Jolla; 8 Sue and Bill Gross School of Nursing, University of California, Irvine; 9 Department of Orthopaedics, Cedars-Sinai Medical Center, Los Angeles, California; 10 Department of Medicine, University of California, San Francisco School of Medicine, San Francisco; 11 Center for Healthcare Policy and Research, Division of General Medicine, University of California, Davis, Sacramento

## Abstract

**Importance:**

Adherence to telemonitoring may be associated with heart failure exacerbation but is not included in telemonitoring algorithms.

**Objective:**

To assess whether telemonitoring adherence is associated with a patient’s risk of hospitalization, emergency department visit, or death.

**Design, Setting, and Participants:**

This post hoc secondary analysis of the Better Effectiveness After Transition–Heart Failure randomized clinical trial included patients from 6 academic medical centers in California who were eligible if they were hospitalized for decompensated heart failure and excluded if they were discharged to a skilled nursing facility, were expected to improve because of a medical procedure, or did not have the cognitive or physical ability to participate. The trial compared a telemonitoring intervention with usual care for patients with heart failure after hospital discharge from October 12, 2011, to September 30, 2013. Data analysis was performed from November 8, 2016, to May 10, 2019.

**Interventions:**

The intervention group (n = 722) received heart failure education, telephone check-ins, and a wireless telemonitoring system that allowed the patient to transmit weight, blood pressure, heart rate, and selected symptoms. The control group (n = 715) received usual care. Patients were followed up for 180 days after discharge.

**Main Outcomes and Measures:**

The main outcome was within-person risk of hospitalization, emergency department visit, or death by week during the study period. Poisson regression was used to determine the within-person association of adherence to daily weighing with the risk of experiencing these events in the following week.

**Results:**

Among the 538 participants (mean [SD] age, 70.9 [14.1] years; 287 [53.8%] male; 269 [50.7%] white) in the present analysis, adherence was lowest during the first week after enrollment but steadily increased, peaking between days 26 and 60 at 69%, or 371 transmissions. Adherence to weight telemonitoring was associated with events in the following week; an increase in adherence by 1 day was associated with a 19% decrease in the rate of death in the following week (incidence rate ratio, 0.81; 95% CI, 0.73-0.90) and an 11% decrease in the rate of hospitalization (incidence rate ratio, 0.89; 95% CI, 0.86-0.91). Adherence in the previous week was not associated with reduced rates of emergency department visits (incidence rate ratio, 0.95; 95% CI, 0.90-1.02).

**Conclusions and Relevance:**

In this study, lower adherence to weight telemonitoring in a given week was associated with an increased risk of subsequent hospitalization or death in the following week. It is unlikely that this is a result of the telemonitoring intervention; rather, adherence may be an important factor associated with a patient’s health status.

## Key Points

**Question:**

Is adherence to weight telemonitoring associated with health care use and death for patients with heart failure?

**Findings:**

This post hoc secondary analysis of a randomized clinical trial that included 538 participants found that an increase in weight telemonitoring adherence in a given week was associated with a significant decrease in the risk of subsequent hospitalization or death in the following week. Adherence was not associated with emergency department visits.

**Meaning:**

Adherence to telemonitoring may be associated with risk of hospitalization and death for patients with heart failure.

## Teaser

This post hoc secondary analysis of a randomized clinical trial of patients with decompensated heart failure assesses whether telemonitoring adherence is associated with risk of hospitalization, emergency department visit, or death.

## Introduction

Heart failure affects millions of Americans and is projected to increase in prevalence.^1,2^ Management of heart failure is complex, often requiring substantial lifestyle changes, including dietary restrictions, medication management, monitoring of symptoms, and other measures. As a result, many patients have difficulty with self-management, which may contribute to high rates of subsequent hospitalization. Heart failure is the most common cause of subsequent hospitalization for adults older than 65 years; more than half of patients are readmitted within the first 6 months after discharge.^2,3^

Telemonitoring interventions seek to help patients with heart failure more effectively manage and avoid exacerbation and its consequences, including subsequent hospitalization and death. Telemonitoring interventions for heart failure typically involve transmission of daily weight values and may also include monitoring of other signs and symptoms, scheduled check-ins with nurses or physicians, or educational components. Evidence for telemonitoring is mixed. A 2015 systematic review^4^ reported moderate evidence for lowering all-cause hospitalizations. A 2018 systematic review^5^ found that telemonitoring in patients with heart failure significantly reduced hospitalization rates at 6 and 12 months.

In a previous study on telemonitoring, Zhang et al^6^ found that patients experienced a lapse in adherence before hospitalization and suggested that this lapse may be an important factor associated with patient outcomes. However, details on this lapse were not provided. In fact, to our knowledge, no studies to date have examined the association between adherence to telemonitoring and patient outcomes. The objective of this study was to describe patterns of adherence to a weight telemonitoring intervention and assess whether a change in adherence was associated with a patient’s risk of hospitalization, emergency department (ED) visit, or death.

## Methods

### Participants and Setting

This was a post hoc secondary analysis of the Better Effectiveness After Transition–Heart Failure (BEAT-HF) trial (NCT01360203), which compared rates of subsequent hospitalization among patients receiving a telemonitoring intervention with those among patients who received usual care after hospitalization for decompensated heart failure. The telemonitoring intervention included heart failure education, monthly telephone check-ins by study nurses, and a wireless telemonitoring system that allowed the patient to transmit daily weight, blood pressure, heart rate, and selected symptoms. Patients were enrolled during a hospitalization for decompensated heart failure. Enrollment hospitalizations could have been the first hospitalization for heart failure or a readmission for previously diagnosed heart failure. Participants were followed up for 180 days after discharge from the enrollment hospitalization. Participants completed surveys on demographic information, social support, health literacy, heart failure–related self-management, depression, and other comorbidities at baseline, 7 days, 30 days, and 180 days. All participants were at least 50 years of age. The BEAT-HF study was approved by the University of California, Los Angeles institutional review board; all participants provided written informed consent. This secondary analysis was covered under the original institutional review board approval because it did not involve additional contact or data collection from or about participants. This study followed the Strengthening the Reporting of Observational Studies in Epidemiology (STROBE) reporting guideline.

Enrollment took place at 6 academic medical centers in California from October 12, 2011, to September 30, 2013.^7,8^ Patients were excluded from the trial if they were discharged to a skilled nursing facility or other contact-intensive environment, if they were expected to improve because of a medical procedure (such as a heart transplant), or if they did not have the cognitive or physical ability to participate in the study activities. In total, 1437 patients were randomized to the telemonitoring intervention (n = 722) or to usual care (n = 715). The primary outcome of this study was experiencing a hospitalization, ED visit, or death in a given week. The complete study protocol can be found in the Supplement. We restricted the current investigation to patients in the telemonitoring intervention who transmitted at least 3 weight values during the study period.

### Statistical Analysis

We focused solely on adherence to the weight telemonitoring component of the BEAT-HF intervention. To explore patterns of adherence, we removed from the denominator days during which the patient was hospitalized, in the ED, or dead. We then examined transmissions by study day, day of the week, and calendar month to describe overall adherence over time.

To examine the association of adherence with subsequent hospitalization, ED visits, and death, we created 1-week intervals beginning the day after discharge from the index hospitalization. These intervals were defined as including an event (hospitalization, ED visit, or death) or being event free. If a participant died during a hospitalization, this event was counted as a death. For a hospitalization that spanned multiple 1-week intervals, only the first 1-week interval was included in the analysis. During the remainder of the hospitalization, the patient was temporarily considered not at risk; thus, those intervals were excluded from the analysis. Intervals after the discharge from the hospitalization were included because the participant was considered to have resumed being at risk for the events of interest. We calculated adherence in event-free weeks as a count of adherent days, which had values of 0 (if no transmissions occurred during the week) through 7 (if weights were transmitted daily). We fit a conditional fixed-effects Poisson regression model to determine the within-person association of adherence with the risk of experiencing an event in the following week. We controlled for the number of previous events (hospitalizations or ED visits) experienced during the study and the number of study months that had elapsed. Number of months in the study was calculated by dividing the number of days in the study by 30.5. The natural logarithm of the interval was included as an offset term. We repeated the analysis restricting to deaths only, hospitalizations only, and ED visits only. We report 2-tailed tests of significance at the *P* < .05 level. Data analysis was performed from November 8, 2016, to May 10, 2019. All statistical analyses were performed using Stata software, version 14.2 (StataCorp).

## Results

A total of 538 patients (mean [SD] age, 70.9 [14.1] years; 287 [53.8%] male; 269 [50.7%] white) transmitted at least 3 weight values using the wireless scale and were included in this study. Of the 538 participants, 351 (65.2%) did not have a college degree. Of those who reported income, 169 (39.4%) reported earning less than $25 000 per year. A total of 49 participants (9.1%) died during the 180-day study period. A total of 257 participants (47.8%) were subsequently hospitalized at least once during the study period, and 95 (17.7%) had at least 1 ED visit. Table 1 gives the demographic and health characteristics of participants.

**Table 1.** Demographic and Health Characteristics of Participants

Characteristic	Finding (N = 538)^a^
Sex	
Male	287 (53.8)
Female	246 (46.2)
Age, mean (SD), y	70.9 (14.1)
Race/ethnicity	
White	269 (50.7)
Black	123 (23.2)
Hispanic	70 (13.2)
Asian or Pacific Islander	35 (6.6)
Other	34 (6.4)
Educational level	
Less than high school	88 (16.6)
High school graduate	263 (49.7)
College graduate	112 (21.2)
Advanced	66 (12.5)
Marital status	
Not married	310 (58.7)
Married	218 (41.3)
Annual household income, $	
<25 000	169 (39.4)
25 000-50 000	110 (25.6)
50 000-75 000	59 (13.8)
>75 000	91 (21.2)
New York Heart Association classification	
I	0
II	99 (22.3)
III	296 (66.8)
IV	48 (10.8)
Hospitalizations during study period	
None	281 (52.2)
≥1	257 (47.8)
ED visits during study period	
None	443 (82.3)
≥1	95 (17.7)
Died during study period	
No	489 (90.9)
Yes	49 (9.1)

Abbreviation: ED, emergency department.

^a^ Data are presented as number (percentage) of participants unless otherwise indicated.

**Figure 1.**
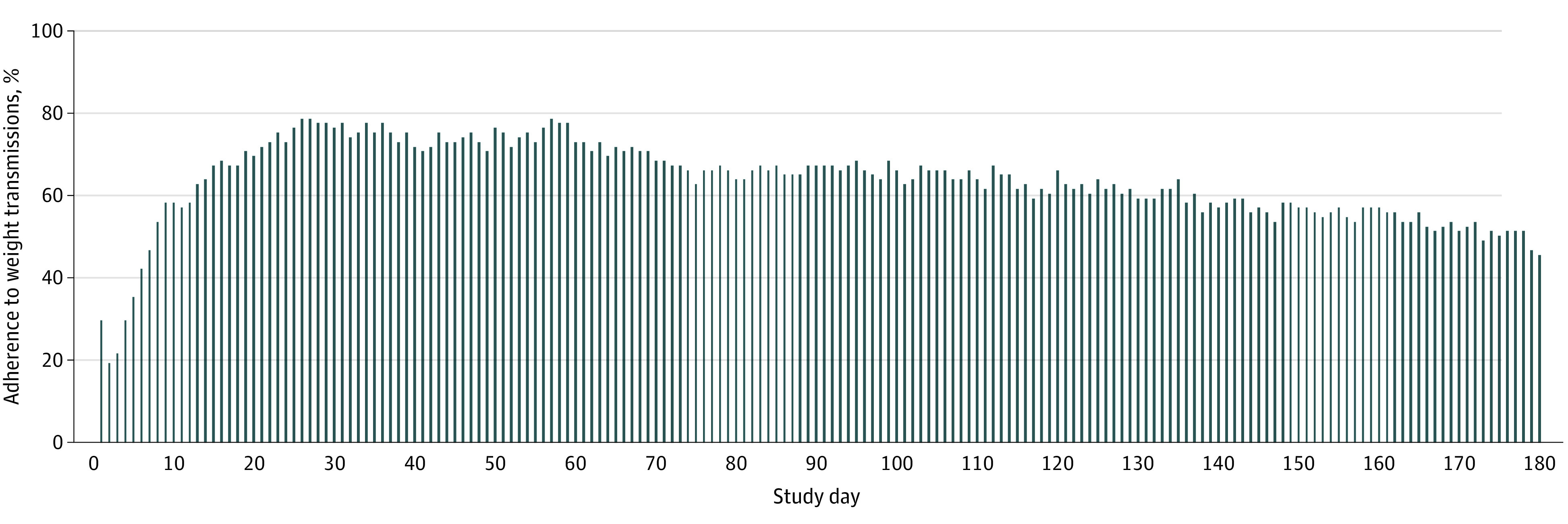
Percentage of Participants Who Adhered to Weight Transmissions by Day in the Study Data are from 538 patients who received the telemonitoring intervention and transmitted at least 3 weight values during the 180 days of the study.

**Figure 2.**
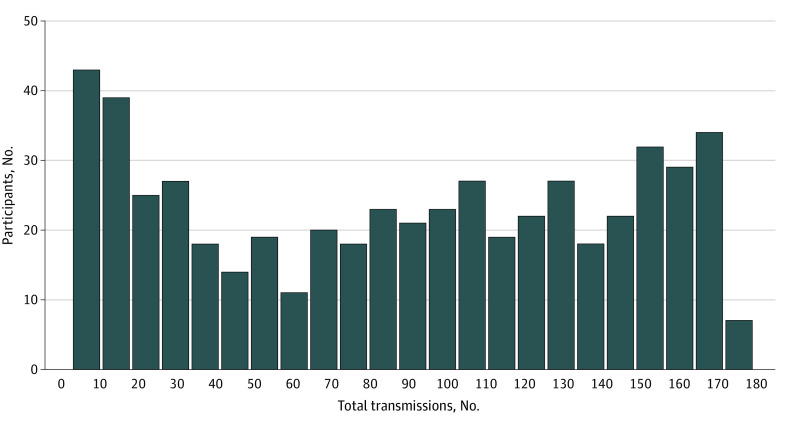
Number of Total Weight Transmissions During the Study Period Data include only 1 weight transmission per patient per day.

**Figure 3.**
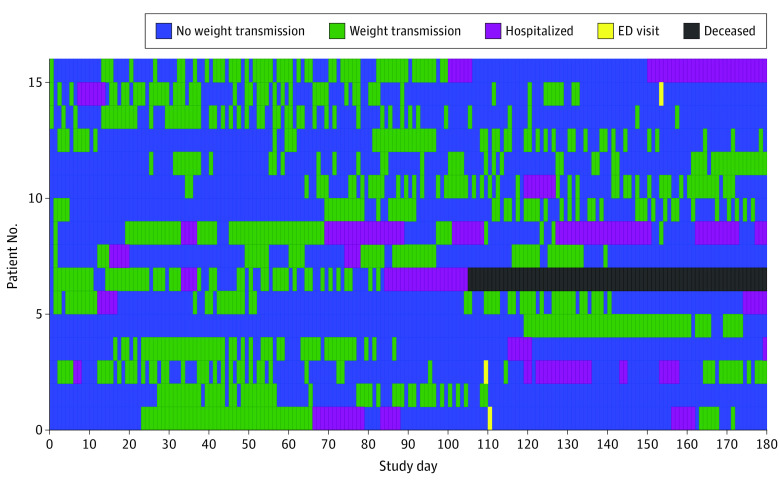
Detailed Telemonitoring Adherence Among Patients Types of days during the study period for 16 patients who had a total adherence of 47% to 53%.

We identified a total of 88 751 person-days as potentially adherent days during which participants were alive, were not hospitalized, and did not visit the ED. Weight transmissions occurred on 47 300 days (53.3%). Figure 1 shows adherence to weight transmissions by study day. Adherence was lowest during the first week after enrollment in the study but steadily increased, peaking between days 26 and 60 and then gradually tapering off so that by the end of the study, mean adherence was still higher than in the first 30 days. On the peak days, 371 patients (69.0%) transmitted a weight value. Participants were less likely to submit a weight value on a weekend day compared with a weekday (69 142 [54.3%] vs 27 698 [50.1%], *P* < .001). Adherence also differed by month, with lower adherence in the winter months; December had the lowest adherence of any month (6821 [46.5%]), whereas August had the highest (9722 [55.8%]). Figure 2 shows the total number of weight transmissions during the study period; although many participants only transmitted weight values several times, participants also commonly transmitted weight values for more than 80% of study days. Figure 3 shows a subset of 16 patients with similar total adherence (between 47% and 53%) but with markedly different patterns of adherence.

Table 2 gives the incidence rate ratios (IRRs) and 95% CIs for events, adjusted for adherence in the previous week, number of previous events, and days elapsed in the study. Adherence to weight telemonitoring was associated with events in the following week. An increase in adherence by 1 day was associated with a decreased rate of experiencing an event by a factor of 0.90 (95% CI, 0.87-0.92; *P* < .001). In other words, a 1-day increase in adherence during the prior week was associated with a 10% decrease in the rate of events. The association was strongest for deaths; a 1-day increase in adherence was associated with a 19% decrease in the rate of death in the following week (IRR, 0.81; 95% CI, 0.73-0.90; *P* < .001). A 1-day increase in adherence in the prior week was associated with an 11% decrease in the rate of hospitalization (IRR, 0.89; 95% CI, 0.86-0.91; *P* < .001). Adherence in the previous week was not associated with reduced rates of ED visits alone (IRR, 0.95; 95% CI, 0.90-1.02; *P* = .15). For all types of events, experiencing previous events was associated with an increased rate of events. In contrast, an increase in months elapsed in the study was associated with a decrease in risk of hospitalizations and ED visits.

**Table 2.** IRRs for Events, Adjusted for Adherence in the Previous Week, Number of Previous Events, and Days Elapsed in the Study (Within-Person Analysis)

Event	IRR (95% CI)	*P* value
All events		
Adherence in previous week	0.90 (0.87-0.92)	<.001
Previous events (any event)	1.26 (1.22-1.28)	<.001
Months in study	0.80 (0.77-0.83)	<.001
Deaths		
Adherence in previous week	0.81 (0.73-0.90)	<.001
Previous events (any event)	1.33 (1.25-1.43)	<.001
Months in study	0.96 (0.81-1.13)	.60
Hospitalizations		
Adherence in previous week	0.89 (0.86-0.91)	<.001
Previous events (any event)	1.26 (1.23-1.29)	<.001
Months in study	0.79 (0.76-0.83)	<.001
ED visits		
Adherence in previous week	0.95 (0.90-1.02)	.15
Previous events (any event)	1.32 (1.25-1.40)	<.001
Months in study	0.84 (0.75-0.94)	.003

Abbreviations: ED, emergency department; IRR, incidence rate ratio.

## Discussion

Our study’s approach is unique in that we examine telemonitoring adherence as a dynamic measure. This approach is in contrast to previous studies^8,9,10,11,12,13,14^ that reported telemonitoring adherence solely as a percentage of study days during which the patient adhered to the intervention. The static nature of this measure is problematic and limits the usefulness of adherence as a predictor in modeling and an outcome in itself. Our examination of patient adherence revealed that adherence was not a static but rather a dynamic measure that often changed within patients over time and had a variety of patterns. As shown in Figure 3, the use of 50% as a cutoff point for adherence shows a subset of 16 patients with similar total adherence (47%-53%) but with markedly different patterns of adherence. The use of 50% as a cutoff point for adherence would miss these differences and has important implications for studying adherence. We believe that adherence should not be categorized as a percentage of possible days or actions but rather as a measure that changes over time. Future research on adherence patterns should explore the possibility of defining adherence phenotypes that describe the way in which people adhere to treatment or monitoring over time. Recent work by Ware et al^15^ has also addressed the importance of examining adherence over time.

Our results align with the results of other studies^15,16^ that indicate that adherence to telemonitoring decreases over time. However, we found that adherence was highest in the second month of the intervention, suggesting that there may be an adjustment period in the first several weeks before adherence peaks. This finding is in contrast to that of a previous study^16^ that reported that adherence was highest at the start of an intervention and decreased after the first several weeks or months. This finding may be in part attributable to posthospital syndrome, which describes increased physiological and psychological stress after a hospitalization.^17^ If patients struggled with poor health or were overwhelmed after the index hospitalization, they may have needed some time to adjust to the new demand of daily monitoring. Alternatively, patients may have encountered initial difficulty setting up the device. Intuitively, our findings indicate that adherence was lower around the winter holidays and on weekends. Because these times may also be when patients are less likely to adhere to other management strategies, such as salt and fluid restriction, implementers of telemonitoring should make a special effort to encourage adherence to telemonitoring during these times.

We found that lower adherence in a given week was associated with increased risk of experiencing a subsequent hospitalization or death in the following week. It is unlikely that this is a result of the actual telemonitoring intervention itself. Rather, this is likely an example of adherence bias or the healthy adherer effect, which refers to the idea that people who adhere to medications or health interventions are typically healthier and more health seeking than are those who do not. This phenomenon has been widely observed in the context of medication adherence.^18,19,20^ Although there is a paucity of literature on this effect in telemonitoring, it is plausible that adherence to weight telemonitoring may be associated with improved patient status. If patients stop adhering to telemonitoring, they may also be failing to adhere to medications or dietary restrictions, which may lead to a heart failure exacerbation. In addition, if patients are feeling weak or frail, this may inhibit adherence to weight telemonitoring and may be a sign of decompensating heart failure. These hypotheses are outside the scope of our study. Nevertheless, our findings have important implications for the design of telemonitoring systems; lack of adherence may suggest a more urgent need for intervention.

Several telemonitoring researchers^6,21,22^ have worked to develop algorithms for predicting subsequent hospital admissions using a variety of measures. A study by Koulaouzidis et al^21^ found that an algorithm using a combination of weight and blood pressure was effective for predicting readmission. However, the authors noted that their predictions were limited by low patient adherence to telemonitoring. Our study supports the suggestions by Zhang et al^6^ and Koulaouzidis et al^21^ that adherence may add value to these predictive models. Our review of the literature did not find any interventions that included adherence to telemonitoring as a factor associated with readmission. However, several previous studies^23,24^ have found medication adherence to be associated with subsequent hospitalization. Wu et al^23^ found that patients who reported that they had missed no doses of their medication in the past week had significantly lower rates of all-cause subsequent hospitalization and death. Another study^24^ found a similar decrease in the risk of subsequent hospitalization among patients with heart failure with improved medication adherence measured by a microelectronic monitoring device in the caps of medication containers. Further research examining the association between medication adherence and adherence to telemonitoring may provide additional insights into the use of these measures as potential predictors of patient outcomes.

### Limitations

This study has limitations. First, we did not take into account how often participants were contacted by study staff after a nonadherent period and what the outcomes of these calls were. It is possible that patients were contacted after a period of nonadherence and instructed to go to the hospital after speaking with the patient about their symptoms. However, it is unknown whether any readmissions could have been successfully avoided, and this explanation does not suffice to explain the association between adherence and subsequent death. Second, we did not have information on the length of stay for patients who were discharged to a skilled nursing facility after subsequent hospitalization. Skilled nursing facility stays may impede adherence to telemonitoring for various reasons, including access to the scale, connectivity problems, and prohibitive policies of the facility. Therefore, this impediment may have resulted in an underestimation of possible adherent days. Third, we were unable to account for days when patients experienced technical problems with the scales. Scale transmission error could have affected our results by underestimating adherent days. We believe that this error, if significant, could have diminished our effect size.

## Conclusions

In this study, lower adherence in a given week was associated with an increased risk of subsequent hospitalization or death in the following week. It is unlikely that this is a result of the telemonitoring intervention; rather, adherence may be an important factor associated with a patient's health status. This study found an association between adherence and subsequent health care use and mortality. We believe that algorithms that alert nurses and physicians when values of weight or other measures are outside the reference range should be adapted to include alerts for changes in adherence to telemonitoring. Such algorithms may help to improve effectiveness of telemonitoring for heart failure. In the future, automated and implantable devices that do not require voluntary effort may be more effective for improving heart failure management.

## Notes

Supplement. **Trial Protocol**
