# Supplementary material for: Association of Adherence to Weight Telemonitoring With Health Care Use and Death: A Secondary Analysis of a Randomized Clinical Trial
Source: JAMA Netw Open. 2020 Jul 10;3(7):e2010174. doi: 10.1001/jamanetworkopen.2020.10174 (PMC7352152; doi:10.1001/jamanetworkopen.2020.10174)
Supplement: Supplement. — Trial Protocol [file jamanetwopen-3-e2010174-s001.pdf]

BEAT - HF

Operations Manual

October 6, 2011

Introduction

This study compares the effectiveness of implementing care transition interventions on reducing variation in readmissions among hospitalized patients 55 years of age or older with heart failure at six medical centers (five University of California Medical Centers plus Cedars-Sinai Medical Center) over an 18-month period with concurrent controls at the same centers. This project compares an adaptation of existing care transition interventions designed to reduce the implementation costs for the hospitals. The study intervention uses a centralized call center, staffed by nurses and supporting all six medical centers, to provide post-discharge structured telephone monitoring of patients, in conjunction with wireless remote monitoring.

Background & Rationale

Recent studies by the Dartmouth Atlas of Health Care have identified geographic variation in hospital resource use and cost at the end of life among elderly Medicare beneficiaries with chronic illnesses. Reduction of variation in resource use and cost is a key focus for saving costs in the U.S. health care system. One potential area of intervention to reduce hospital resource use and cost variation is readmission rates, and interventions designed to improve the care transition period after hospital discharge have been shown to reduce readmissions and potentially improve morbidity and mortality at the patient level. However, no studies have demonstrated if care transition interventions would result in reductions in variation among hospitals on resource use or health outcomes. In addition, interventions that improve care transitions may be cost-effective at a societal level, but have not been widely disseminated due to implementation costs at the hospital level.

This comparative effectiveness project builds on our prior work examining variation in resource use and mortality among the five University of California Medical Centers plus Cedars-Sinai Medical Center for elderly Medicare beneficiaries hospitalized with heart failure. This work will begin to bridge the current gap between quality improvement research and studies of variation in care, and also provide an opportunity to compare the effectiveness of an existing care transition intervention with an approach that utilizes new technologies. Current studies of variation rarely have the clinical or organizational data to suggest ways to reduce variation between sites, and quality improvement work is often focused on changes at the patient or specific institutional level but not across institutions. Although existing care transition interventions are effective, they have yet to be widely disseminated due to their costs; telemedicine and centralized telephonic interventions that can be simultaneously implemented across a heterogeneous set of hospitals hold promise for potentially reducing these costs.

Trial Objectives

1. Compare the effect of implementing the care transition intervention with concurrent controls on variation in readmissions among patients 55 years of age or older who have been hospitalized with heart failure at the six medical centers.
2. Examine the change in variation over time in readmissions and mortality among hospitalized Medicare beneficiaries with heart failure at the six medical centers.
3. Compare the health benefits and costs of the care transition intervention

Trial Procedures

Inclusion Criteria
Patients 55 years of age or older, who are hospitalized with a principal diagnosis of heart failure.

Exclusion Criteria

- If a patient is admitted from a Skilled Nursing Facility or would be expected to be discharged to such a facility. Looking in the past medical history of the admission note helps to identify that a patient is coming from a SNF.

- History of transplantation or being evaluated as a future transplantation candidate. Looking into past medical history would help to identify if the patient has had transplantation in the past. A patient may be on the waiting list for a transplant at one of the six study medical centers. Patient and/or family members could be asked for confirmation. Please note that this includes any transplant – whether heart, lung, kidney, etc.

- Dementia – If a patient has dementia, he or she will be excluded from the study. This can be found through the admission note or past medical history. In addition, a dementia screener will be administered as part of the consent process.

- Chronic dialysis patients. Review of admission notes or past medical history could help identify chronic dialysis patients. Patient and/or family members could also be asked for confirmation. Another tip would be the presence of a fistula or a dialysis catheter. Please note that patients with peritoneal dialysis could be included in the study but there are few such patients.

- Lack of a working landline or a reliable T-mobile coverage. Patient's residence should be under a reliable T-Mobile network coverage (as ascertained by T-mobile coverage area maps) if they do not have landline.

- Inability to use the intervention equipment, such as the weight scale. For example not being able to stand on the weight scale, or if a patient weighs more than 390 pounds (exceeding the tolerance of most devices) he/she will be excluded from the study.

<u>Various Considerations</u>:

- If there is doubt or question about including or excluding a patient, the site nurse should contact the site PI or the site study cardiologist and cc Dr. Ong on their email.
- Patients who speak English or Spanish are included.
- Patients who are admitted to the hospital with pacemaker dysfunction could be included.

- Patients who are hearing impaired or visually impaired but can demonstrate to the site nurse that there is someone to help them with study devices are included. It would be up to the nurses' discretion to make this decision. For example it would be fine if a hearing impaired patient has someone who can answer telephone calls at any time, or if a visually impaired patient has a part time caretaker who can assist with the BP cuff and question pod on a daily basis (the scale has a voice option).
- Patients with coexistent arrhythmia can be included in the study.
- Patients who are transferred in from another site are eligible for participating in the study.
- Patients should be informed that if they travel outside of California or out of the country (e.g., Mexico) for extended periods of time, the call center nurses or research staff might not be able to provide them services and they might need to be withdrawn from the study depending on the site PI's decision. The California nursing license is not valid out of state. Patients traveling or residing outside of the US will need to be informed that they may incur charges for daily calls from their devices and they will need to provide us with a local number so that the call center nurses can make their calls without incurring international call charges.
- All hospice-bound patients are excluded from the study.
- Patients who cannot identify a usual source of healthcare are excluded. The usual source of healthcare could be a free clinic, where the patient might not be able to identify a specific provider.
- Patients who are on United Health Care heart failure remote monitoring program (or similar programs) are excluded from the study (both control group and intervention group).
- Marginally housed and homeless individuals are excluded unless they are able to handle housing and the maintenance of the technology components and could comply with all aspects of the intervention. Nurse's discretion and the judgment of those who have approached these patients is important here.
- If a patient cannot comply with the intervention or is unable to use the intervention equipment, he/she will be excluded from the study.
- All surgical valvular patients except for those with incidental disease will be excluded from the study.
- All AMI patients except for those with demand ischemia will be excluded from the study. AMI is defined as cardiac enzyme (trop, CKMB) rise and fall plus
    a) ischemic symptoms
    b) development of Q waves
    c) ST elevations/depressions
    d) PCI

  Documentation of demand ischemia is the chart will count for demand ischemia. If the case is still is unclear, the site nurse should check with the inpatient attending for confirmation.
- All patients receiving PCI will be excluded from the study.

Identifying Potential Patients
As indicated in the inclusion criteria, all patients admitted to the hospital who are 55 years of age or older, and who are admitted for treatment of heart failure should be considered as potential subjects for this study.

Every day, each site will obtain a list of recently admitted patients during the prior day (or prior three days on Mondays). This list is generated in different ways at the different sites, and may be provided by Admission, ER, Cardiology Service Director or from data generated by the Quality Management Services.

This list should include basic information such as name, age, principal diagnosis or symptoms on admission, and MRN. Based on this list and after using medical record number (MRN) and date of birth (DOB) to exclude patients who have been previously approached by the team (either enrolled, excluded, or declined), a preliminary list of potential HF patients will be developed by focusing on the principal diagnosis. All patients with presenting symptoms consistent with HF should be considered for further evaluation.

The following keywords could be used to find patients whose initial principal diagnosis is not HF, but still could be a potential primary case of heart failure: shortness of breath, edema, dyspnea, weakness, or fatigue. Other keywords that might also include patients whose initial principal diagnosis is not HF, but still could be a potential primary case of heart failure: arrhythmias, unstable angina, pneumonia, chest pain, and altered mental status. For these cases that might be HF, the study nurse will need to check the admission note or with the attending physicians to confirm if the suspected patients on the list have HF or not as their principal diagnosis.

Since some HF patients will stay in the hospital for just a short period of time, study nurses should try to identify HF patients admitted the prior day(s) as soon as possible to take necessary steps for enrollment into the study.

Previously Enrolled or Declined to Participate Patients
Patients who have been approached in the past for the study and either enrolled or declined to participate, should not be re-approached. As the enrollment software (PIweb) includes name and medical record number (MRN) of the patient, it can be used to determine whether a patient has been approached before.

If a patient has not been approached on a prior admission (for example if a patient was a weekend admission and was discharged before could be seen), it is ok to approach.

If a patient is identified as being previously enrolled in the study, the study nurse will send an email to the site PD with study ID and new admission date. The site PD will then notify the UCLA PD with this information, who will then relay the information to the overall principal investigator and the data analysis team. If the patient is an intervention patient and the

timeframe is within the six months of enrollment, the call center will be notified.

Screening evaluation
The admission notes of all heart failure patients on the finalized daily list will be reviewed by study nurse for exclusion criteria. Patients being admitted from a skilled nursing facility will be excluded. For transplant patients, this is determined by checking the past medical history for receipt of a transplant, if the admission note states the patient is being admitted for transplant evaluation, or if the patient is on the waiting list for a transplant at one of the six study medical centers. The admission note will also be checked for information indicating the patient has dementia.

For the next part, oral consent will be used for screening procedures for the below four subsequent specific items that may exclude an individual from participation: chronic dialysis, lack of a working landline or T-mobile cell service, inability to use the intervention equipment, or dementia. Patients will be approached by the study nurse at each site to complete a brief dementia screener, determine if they are a chronic dialysis patient, and asked whether they have a working landline or T-mobile cell service or whether they can use the intervention equipment. For individuals for whom there are no positive answers on the dementia screener and who are not chronic dialysis patients, can use the intervention equipment (e.g., are not over 390 pounds), and have a working landline phone or T-mobile cell service, consent procedures will be initiated.

Informed Consent
At this point, eligible patients will be informed about the study and consent will be requested. The patients should be given approximately one day to decide whether or not they would like to participate in the study. The site nurse will meet with the prospective participants/families to review the consent document(s) and/or provide an oral explanation of the study. Individuals will be given the opportunity to ask questions and/or consult with family and/or friends before making a considered decision about whether or not to participate in the study. Signed consent and HIPAA forms will be obtained from the research participant or Legally Authorized Representative. Signed consent means research participants will be asked to sign and date a written consent form. A copy of the consent and HIPAA form will be kept in patient's medical record and another copy would be placed in the study binder that will be sent home with the patient.

For non-English speaking patients, if the site nurse does not speak the patient's language, the site should use an interpreter service (phone or in-person).

The PDs in each site will keep all patients' signed consent and HIPAA forms in the source document binder in a secure place (locked office, locked drawer). Scanned copies of the signed consent and HIPAA forms will be sent by encrypted email by PDs to the UCLA PD as PDF files on a weekly basis for back up purposes.

Patients will not need to sign the California Bill of Rights since the UCLA IRB does not require a signature for this document and all sites have deferred to the UCLA IRB for the study approach.

Enrollment

Once the patient has consented to the study, they are considered enrolled in the study. If a patient is enrolled in the study and at some point did not continue, he/she cannot be re-enrolled.

The patient will be assigned a study ID at this point. Each site will generate its own patients' study IDs. The study ID is a unique 6-digit code that starts with a number between1 to 6 which corresponds to the institution to which the patient is admitted (1=UCD, 2=UCI, 3=UCLA, 4=UCSD, 5=UCSF, 6=CSMC). The PIweb system will not let the user to generate a study ID which has been previously created, so there will be no chance of generating duplicate numbers.

PIWeb

There is a central enrollment-tracking database (PIWeb), which will contain information important for study nurses, call center nurses and survey research staff. At the beginning of the enrollment in PIWeb, the MRN and the hospital the MRN belongs to is entered in the PIWeb. A unique study ID number will be assigned to each patient by the study nurse who is enrolling the patient. PIweb also contains the procedures for randomization and for communication regarding patients randomized to the intervention.

Once a patient is enrolled, regardless of when the discharge time would be, we need to continue moving forward with study activities. It may be helpful for the site nurse to check with the floor nurses before attempting enrollment to find out if the patient is expected to be discharged shortly.

We should avoid making notes in the patient's medical record that he/she has been enrolled in this study since that may skew hospital provider (physician and nurse) perceptions of the control vs. intervention patients.

Best telephone number to use

All patients need to be asked this information, as both control and intervention patients will receive calls from the survey staff after discharge. This information will be entered in PIWeb for all study patients.

New York Heart Association Classification (NYHA)

Heart failure patients experience various symptoms such as limitation in daily physical activity, fatigue, palpitation, or dyspnea. Based on existence and severity of the symptoms, patients are classified into 4 groups, and the classification is recorded in PIWeb for all study patients.

Class	Patient Symptoms
Class I (Mild)	No limitation of physical activity. Ordinary physical activity does not cause undue fatigue, palpitation, or dyspnea (shortness of breath).
Class II (Mild)	Slight limitation of physical activity. Comfortable at rest, but ordinary physical activity results in fatigue, palpitation, or dyspnea.
Class III (Moderate)	Marked limitation of physical activity. Comfortable at rest, but less than ordinary activity causes fatigue, palpitation, or dyspnea.
Class IV (Severe)	Unable to carry out any physical activity without discomfort. Symptoms of cardiac insufficiency at rest. If any physical activity is undertaken, discomfort is increased.

Table Extracted from Heart Failure Society of America website at http://www.abouthf.org/questions_stages.htm

Baseline Survey

Once consent is obtained and the patient is enrolled, the study nurse will administer the baseline survey. The baseline survey is administered by the study nurse using a web-based survey system software while the patient is in the hospital and before randomization is performed. A paper copy should also be provided to patients to assist with completion. Both intervention arm and control arm patients will also be evaluated by baseline survey and follow-up surveys after discharge. Each patient will also be contacted by telephone within 7 days, 30 days and 180 days post-discharge by the survey research staff for other remaining surveys. The survey information will be stored on a secure UCLA server, and only the analysts and the data safety monitoring board (DSMB) will have access to it.

Components of Baseline Survey:

1) REALM-R -- Rapid Estimate of Adult Literacy in Medicine, Revised

To assess how familiar the patient is with some basic medical words.

2) Self-Care of Heart Failure Index

To assess the patient's ability to care for their heart failure.

3) Minnesota Living with Heart Failure Questionnaire

To assess how much the patient's heart failure has affected their life.

4) Lubben Social Network Scale

To assess the patient's social relationship with friends and family.

5) Medicare Care Questions

To assess how much family members and friends are involved in assisting the patient with health care.

6) Geriatric Depression Scale

To assess potential depression.

7) Advance Directive Question

8) Demographics
9) WHO HPQ - Absenteeism and Presenteeism Short Form
These are questions about patient's work in order to assess the impact of missed days from work.

TIBI
The TIBI (Total Illness Burden Index) questionnaire will be given to patients in both arms of the study while the patient is in the hospital, in order to further assess comorbidities. Patients will be given a hard copy of the TIBI to fill out on their own, but will be assisted upon request.

Randomization
Once the baseline survey, TIBI, NYHA classification, and the consent process is completed, the patients will be randomly assigned to either the intervention group or the control group using a web-based computerized random number generator in the randomization section of PIWeb. As randomization occurs upon enrollment, those patients randomized to the control group should not receive additional services beyond usual care that will be provided to the patients in the intervention arm.

Post-Randomization Steps – Control Group
The control group will receive the standard of care that is normally provided by the admitting hospital.

Please note:

- No information about these subjects will be sent to the call center.
- NYHA classification will be determined for control group patients and will be entered into PIWeb.
- A contact number and best time to call will be taken from control group patients to be used by survey group for their phone calls after discharge and will be entered into PIWeb.
- No letter will be sent to physicians whose patients are assigned to the control arm of the study.

Following discharge, control patients will be contacted by the survey team during the first week after discharge, 30 days and 180 days after discharge.

Patients in the control arm will receive a control patient study binder to help remind them that they are participating in the study and to remind them that they will be contacted for the over-the-phone at 7, 30, and 180 days post-discharge surveys.

Content of BEAT-HF Binder for Control Group Patients

- Consent
- HIPAA
- Study Information (e.g., Survey Calls)

- Space for site specific patient education

If there is any educational material at a site, the control group patients should receive it from their regular nurses. If control group patients ask for further educational material, site study nurses shall refer them to their regular nurses.

Post-Randomization Steps - Intervention Group

Site study nurses will communicate essential information about enrolled intervention patients through PIweb as they go through the intervention procedures. Call center nurses will have the capability to look into PIWeb daily and see all new subjects that have been enrolled and randomized into the intervention arm. Information that is reflected in PIWeb will make the hand-off process as efficient as possible, will help the call center nurse quickly know what education each patient has received and which topics should be reemphasized, and will avoid duplication of effort and manual entry of data.

The following information is captured in the communication section of PIWeb:

- General information about patient such as name, age, MRN, date of discharge, preferred language, phone number, best time to reach, secondary contact number, etc.
- Information about barriers to care and learning
- Completion or not of each of the modularized teaching items, the level of patients' understanding in each one, and whether a caregiver has also been trained or not.
- Patient's providers' information
- Ideal Life serial number for pod, cellular adapter cuff, and scale; and whether the patient has taken the device from hospital or asked that the device be shipped

This information will be gathered and recorded in the system by study nurses during the enrollment, randomization, and post-randomization steps.

We need to be very vigilant that the patients receive the standard of care that should be provided by the regular nurses, and that they are not overlooked by the floor nurses just because they have been assigned to the intervention arm. The study nurse at each site will work with each patient following enrollment and will go through the Education Checklist to ensure the patient is educated about heart failure, self-management, and use and purpose of the Ideal Life equipment.

BEAT-HF Binder for Intervention Patients

Every patient who gets enrolled in the study will receive a BEAT-HF booklet, but the intervention patient binder has more content than the control patient binder. The intervention patients will have the Ideal Life related stuff and the educational material prepared for the intervention group in their booklet.

Content of BEAT-HF Binder for Intervention Group Patients

- Consent
- HIPAA
- Study Information (e.g. Survey Calls)
- Call center nurse names (with photo)
- Educational material
    - Modified UNC booklet
    - Fluid management
    - ZONES
    - Physical Exercise
- Self-Management Examples
    - Weight management (Calendar)
    - Medication List
- Ideal Life Device Information
    - Serial numbers (Cellular pod, cuff, pod, scale)
    - Manual
- Notes
    - Discharge date
    - Discharge weight
    - Follow-up appointment
    - Doctor's name and phone number
    - Questions for my doctor
    - Hospitalization History

Site study nurse will walk the patient through the booklet and explain and instruct patient on the topics page by page.

Identifying the Call Center Nurses for Each Patient

In order to have a more personal handoff from study nurses in the hospital to the call center nurses who will be managing their post-discharge care, each patient will be paired with a primary call center nurse based on the institution at which they were hospitalized. Upon discharge each patient will be given a sheet in their study binder with the name and photo of the paired call center nurse. The information of other call center nurses would also be provided to each patient on the same sheet of paper, as there will be occasions, e.g., trigger calls or days off, that a call center nurse other than the patient's usual call center nurse may need to contact them

Barriers to Care

One of the responsibilities of the study nurse is to talk to patients to understand if there are any barriers to care. Some barriers that could potentially impede patients from acquiring appropriate care include:

Language barrier

Emotional limitations
Physical limitations
Cognitive limitations
Financial
Lack of motivation
Cultural factors
Transportation barriers
Health literacy
Other

Indicating barriers to care for each individual will help the healthcare staff and call center nurses to focus on resolving those issues. This information will be entered into PIWeb by the site study nurse so that it is available to the call center nurses.

Education Checklist
Site study nurses have a checklist of education topics in PIweb that should be covered before an intervention arm patient is discharged. The overall goal is to complete patient-oriented education, not completing the checklist. The checklist should be used to document what patient-oriented education was completed. The sections in the checklist cover the following items:

- HF basics -- diagnosis & recognizing symptoms
- Daily assessment
- HF medications
- Low-salt eating plan
- Fluid intake
- Physical activity
- Follow-up care
- Study process & equipment

There are specific key elements within each of the above components that are included in the Communication Form. The Communication Form also allows the Study Nurse to indicate who received the education – the patient, the family, or both. It might be someone else in the family who does the cooking or fills out the medication bottles. Call Center Nurses will have full access to the notes that site nurses put in this checklist through PIWeb. The patient's receptiveness to change should be noted and relayed to the CCN, because this may affects their potential trajectory post-discharge.

Heart Failure Diagnosis and Recognizing Symptoms
It is important to educate patient in a simple and comprehensive way. For this purpose the educational material will be provided to each patient in terms of a booklet which will help the study nurse to structure teaching to patients.

The main goal is to educate the patient about his or her diagnosis, and key lifestyle measure using teach back approaches.

Here are examples of some teach-back questions:

1) *What is the name of your water pills or diuretic?*
2) *What weight gains would you report to yourself? How do you weigh yourself?*
3) *What foods have you been told to avoid? Name high sodium foods.* (Emphasis should be placed on a low salt education).
4) *What changes in yourself would prompt you to call the doctors?*

Review the appropriate steps for what to do if a problem arises
The patient should be educated by the study nurse about the appropriate steps for what to do or whom to first call if a problem arises.

The study nurse will discuss with patient:

- A specific plan of how to contact their primary heart failure provider, which could be a cardiologist or a primary care provider depending upon who the patient identified during the enrollment process
- What constitutes an emergency and based on symptoms when to call a doctor versus when to go to emergency room

Medication teaching
The study nurse will educate patient regarding various heart failure medications and the indications of each category:

- What medications to take
- Review each medication's purpose
- Review dosage and important side effects to watch out for
- Make sure patient has a realistic plan about how to get the medications (use teach back approaches)

Identify key outpatient providers
The outpatient primary heart failure provider is determined for the patient prior to discharge. Most of patients would have already a primary heart failure provider who might be a primary care physician or a cardiologist or other specialist. If the patient has a primary care physician and a cardiologist, the preference is to use the cardiologist as the patient's primary heart failure provider.

Appointment, Phone Calls, and Surveys
Subject will be asked whether there is a preference in terms of day and time to be called by call center nurses. A sheet with name and photo of three call center nurses will be given to patients to familiarize them with the nurses who will call them after discharge for a better hand-off.

The study nurse will work with each patient to:

- Encourage attendance at post-discharge appointments for clinician follow-up
- Elicit input from the patient on the best time and date of the appointment
- Make sure the patient understands the importance of such services
- Confirm the patient knows where to go and has a plan about how to get to an appointment
- Review transportation options and other potential barriers that might keep patients off their appointments.

The study nurses shall not schedule an appointment for the primary outpatient provider. The goal is to encourage and empower patients to actively inquire and achieve their own healthcare needs. If patients inform the study nurses about a pre-scheduled appointment with their primary HF provider, or the post-discharge appointment has been set, such information could be entered in the Communication Form in the PIWeb.

Equipment
The study nurse will educate each patient on use of the wireless remote monitoring devices. Study nurses will instruct patients prior to discharge on how to use the weight scales and BP cuff, and teach the patient on how to use the communication device (i.e. data transmission, text message receipt and sending). The focus should be on relating the self-care they have been educated on with use of the equipment. Patients and/or caregivers will be asked to demonstrate proficiency with using the equipment prior to discharge. Teach back methods should be used to ensure that the patient knows how to use the equipment. Patient should demonstrate putting on the BP cuff, standing on the scale, and going through the box symptom questions. An Ideal Life device user guide with simple language and large font is included in the intervention patient binder.

Equipment: Connectivity
The equipment transmits best via landline. If there is no working landline, the equipment could be connected to the provided cellular connector for the purpose of transmitting the results. However, the cellular connector uses T-mobile coverage, and the T-mobile coverage map should be checked if the patient reports not having a landline. Unfortunately cellular connectivity could potentially be disrupted by other wireless devices in the patient's home like multiple Bluetooth devices. Patients should be warned about the possible interference, one suggestion would be to have the patient turn off other wireless devices when they conduct their daily transmission.

- Landline – the first choice
- Cellular Connector – the second choice, requires T-mobile network coverage, also potential connectivity disruption by means of other existing wireless devices.
- No Ethernet

Equipment: Troubleshooting/Failure

In the event of equipment malfunction at the site prior to being sent home with a patient, the Project Director will contact Ideal Life at 1-800-611-2660 for troubleshooting assistance. If equipment is determined to be malfunctioning, complete an Equipment Failure Report and return the equipment to Ideal Life.

Patients will receive prior to discharge the biometric remote sensor devices (weight scale and a BP cuff to measure BP and heart rate), and the communication device, unless they ask for these to be mailed home.

Equipment: Going Home

The serial numbers of scale, cuff, cellular adapter, and pod given to the patient must be entered in the patient's PIWeb system by the site study nurse. These numbers allow the call center nurses to track patients, after entry into the Ideal Life website.

Preferably each patient will take his/her equipment from hospital to home at the time of discharge. However, some patients might ask to receive it by mail. Equipment will not be sent before the patient's discharge. The site study nurse should confirm patient's address and preferred time of delivery within the Communication Form. The devices will be sent by Fedex overnight service (for tracking purposes) to patient's address from UCLA. The device serial numbers will be entered by UCLA.

Equipment: Site Storage

We should strive to keep approximately 10 sets of devices at each site. When the number of devices drops below 5, the site PD should be notified so that they can communicate back to UCLA for delivery of more devices. Equipment should not be placed in extreme climates (too hot, too humid) or in direct sunlight or dust. Equipment should be tested upon receipt to ensure they are working properly. If needed, these should be cleaned as noted in the section regarding isolation patients.

Equipment: Isolation Patients

Some patients are in isolation. Please follow the below protocol to minimize risk of infection:

1. All equipment items should be cleansed with Clorox (or equivalent)wipes before leaving one patient's room or hospital, regardless whether patient has been in isolation or not.
    a. Clearly identify and separate areas for clean and used equipment. Maintain storage area cleanliness.
    b. Equipment should be cleaned by wiping down with a cloth dampened with solution of water and mild detergent, or use mild antibacterial wipes. Glass cleaner can be used to clean smudges on plastic or glass surfaces.
2. For isolation patients, in order to minimize contact with items of equipment, consider using a cover where possible (e.g. baggie over scale) if the patient will not be taking the equipment home with them.

Unfinished Pre-Discharge Activity

If there is an unexpected or premature discharge before the study nurse activities are completed, this should be documented by the study nurse in the nurse communication area in PIWeb. The call center nurses will then know what was actually covered during the hospitalization, and can provide patients with additional education or information as needed (e.g., how to set up the Ideal-Life devices). However, we expect most patients to complete most, if not all, of the study nurse activities prior to discharge.

Letter to Primary Heart Failure Provider

Upon randomization to the intervention arm, the Project Director will send a letter to the patient's primary heart failure provider to let him/her know about the trial and that a new patient has been enrolled in the intervention arm of the study. A summary of the study and the default biometric monitoring parameters will be included in the letter and the provider is requested to let us know if he/she would like to modify the parameters for any given patient. The letter will also indicate the method by which we plan to contact the provider for urgent and for non-urgent matters, and will also request that we be notified if a different method is preferred. Every site will use the project standard letter, but it will be sent from the local site PI's email address.

Intervention Post-Discharge Activities

Remote Monitoring

Following discharge, patients will be asked to transmit automated biometric information and symptoms daily to the centralized call center using the communication device. Patients will be instructed to turn on communication device upon waking up in the morning and to turn it off before they sleep at night. As soon as the patient's personal communication device is switched on, it will remind the patient to perform their daily "Health Check" (e.g. obtain daily weight and vital signs). After being prompted to respond to a series of symptom questions related to their HF status and general health, patients will capture their weight and vital signs using the scale and BP cuff. Information from the remote monitoring system will be automatically downloaded to a secure Internet site for review by the call center nurses, with individuals flagged who have "variance triggers" from daily symptom reports or biometric data that are outside specified parameters (such as a weight gain of greater than 3 pounds in one day or 5 pounds in one week). At the end of six months, patients will be asked to return the monitoring equipment to the medical center using preaddressed packaging with pre-paid postage.

Call Center

Call center nurses contact the intervention patients preferably within 3 days of discharge to reinforce patient education, medication and follow-up plans and to conduct problem-solving if necessary. Prior to discharge, all patients are informed to expect a follow-up call within 3 days of discharge. Names and photos of call center nurses will be printed on a sheet of paper and will be given to patient within the intervention patient binder. The call center nurses have access to the PIWeb site (contains Communication Form completed by site study nurses for all patients enrolled into the study), and the patient's EMR with latest note in the patient's medical record. Call center nurses will also conduct trigger calls, scheduled weekly calls during the first month, and scheduled monthly calls through six months. The Call Center Nurses should act as a medical coach and not as a pass-through where it makes sense for them to make a clinical decision. Calls might be recorded for quality assurance purposes. The call center will operate also during weekends and holidays, but generally only for responding to trigger calls or, if not avoidable, a scheduled outbound call.

The goal of these calls is not to replace routine management, but to facilitate management. We want to empower intervention arm patients to be proactive in calling their provider when they need to, and for the call center nurse to provide necessary information back to the HF provider. For example, it is not call center nurses' responsibility to schedule office appointments for subjects. Instead, patients would be encouraged to contact their physician directly and make the appointment themselves (e.g, if needed, coaching patients on what to say when calling the physician's office). Patients are encouraged to contact their regular care source when needed. Call center nurses could contact other study staff in different sites when having difficulty in contacting provider.

First Follow-up Call

The goal is that first call be placed in the first three days after discharge. We are allowing a three day window before or after the third day after discharge. If the first call happens in the

second week, (day 8 through 14), it would be considered the second call out of 4 weekly calls (in this case the first call has been skipped). We will try to minimize stacking post-discharge calls (standard site calls, intervention calls, and survey calls). The expected duration of first call will be approximately 90 minutes in length.

During the first follow-up call the call center nurse will assess any potential problems with care transitions. Any identified issues, medication or otherwise, will be relayed to the primary HF provider, or if different, the primary care provider or provider who will first see the patient following contact. If the patient is having trouble scheduling timely outpatient care, the call center nurses will work with the patient to solve how to set an appointment with the outpatient primary HF provider.

It is recommended to evolve the relationship by asking questions like "is there anything about your health we can help you with today?" Such questions will help demonstrate to the patient that the call focuses on their agenda. Patients should also be asked what is the best day and time to reach them for next calls. This gives patients a sense of empowerment and establishes some sort of buy in for them. Patients are more available when asked for this information.

The following checklist would be used during the first call:

- Review/Reinforce patient education
- Review follow up plans (CCNs will have the ability to tap into EMRs to see if follow-up plans like appointments may have occurred)
- Review specific medications within classes that are important treatments for heart failure (including not using COX-2 inhibitors); call center nurses will ask patients to bring their medications to the telephone to review them and address medication-related problems.
- Review/Reinforce troubleshooting plans (e.g., who should be contacted and under what circumstances)
- Troubleshooting HF and non-HF issues, especially HF issues.
- Make sure patients are adhering to their treatment plans.
- Attend to problems or potential problems as efficiently as possible.
- Reinforce the use of the remote monitoring technology.

If a patient asks a question that call study nurses do not know the answer, they should encourage the patient to call his/her provider. It is discouraged that the nurses call the provider to help the patient with his/her question.

Weekly calls

Patients will subsequently be called at a minimum on a weekly basis for a total of at least four telephone contacts during a 30-day period. However, call center nurses may increase the total number of calls during the 30-day period as deemed necessary.

The 4 weekly calls are more or less similar in terms of discussions, but the emphasis would be on those issues that patient needs more education and guidance on (e.g., troubleshooting). It is

estimated that the duration of each weekly call be around 1 hour, but will be dependent on the course of patient's conversation with the call center nurse.

The following checklist would be used in weekly calls:

- Review/Reinforce patient education
- Review follow up plans
- Review/Reinforce troubleshooting plans
- Troubleshooting HF and non-HF issues, especially HF issues.
- Make sure patients are adhering to the intervention.
- Make attention to problems or potential problems as efficiently a possible.
- Reinforce the use of the Ideal Life technology.

Monthly calls
After the 30-day period, call center nurses will contact the patients on a monthly basis up through six months after discharge. These monthly calls will not be as intensive (e.g., will not explicitly go through each medication as described earlier) as the calls during the first 30-days period, but will be problem-oriented and will provide guidance on any HF management issues raised by the patient and/or caregivers. Reinforcing the use of the Ideal Life technology should be an element of each call.

HF Documentation Tool
The HF Documentation Tool has been developed by call center nurses as a template for the software that will structure their patient calls, support their documentation, and enable them to share information about a patient within their group. It is not intended to be an assessment that will be completed on each call. It is intentionally comprehensive, but not all of the sections will be relevant to all patients on all calls. This template will help to guide their calls and enable them to document and – if a different person has to call the patient back – they can share information amongst themselves.

Inbound Calls
Patients are not encouraged to call the call center. There will be a voice mailbox in place at each site for patients who call and leave messages surrounding non-urgent issues; however, the outgoing message on these voice mailboxes will inform the patients that these mailboxes are checked on an infrequent basis, at most once daily and on weekdays only. Patients are not encouraged to call these numbers, but we understand that some patients may capture the call center phone number on caller id and choose to call the number to try to reach their call center nurse. While all outgoing calls from the call center nurses will be coming from the centralized call center at UCLA, our phone system allows for a local area code phone number (e.g., 415 for san Francisco) to be programmed for display on the call recipient's caller id. So patients at each site would see a "local" phone number and if they chose to call it, would then reach the local voice mailbox that has been set up at each site for purposes of the study. The local voice mailbox will be checked periodically by the local site PD, and the PD will send an email to the

call center nurse so that they can return the call, if needed. Such calls will be entered into the call center nurse system similar to any other trigger call.

Non-English Patients
For non-English speaking patients, if the site nurse does not speak the patient's language, the site should use an interpreter service (phone or in-person). Similarly, the call center nurses can place calls through a third party on TDD line to communicate with patients who have hearing or speaking problems. TDD allows such patients to read the conversation on a small monitor of their specific telephone gadget and reply back by typing instead of speaking.

Parameters and Trigger Alerts Protocols
Call center nurses have an important role in observing the results of daily measurements and taking necessary actions if they encounter a trigger alert or realize that a patient has become symptomatic. There is a set of default thresholds for various biometric parameters that could trigger an alert, and potentially a call to the primary HF provider. Primary HF providers are informed of the default parameters and are given the opportunity to change them up front by contacting the local Project Director, whose name and contact information is provided in the letter sent to physicians upon enrollment of one of their patients in the intervention group.

The table below depicts the parameters that will prompt a call from the call center to the patient:

**Biometric Parameter**	**Threshold**	
SBP	SBP < 90 mmHG	SBP > 160 mmHG
Heart Rate	HR < 50	HR > 100
Weight	Daily gain > 3 Pounds	Weekly gain > 5 Pounds

The table below depicts the parameters that will prompt a call from the call center to the patient as well as to the primary HF provider:

**Biometric Parameter**	**Threshold**	
SBP w/ Symptoms	SBP < 90 mmHG	SBP > 160 mmHG
SBP w/o Symptoms	SBP < 80 mmHG	SBP > 170 mmHG
Heart Rate w/ Symptoms	HR < 50	HR > 100
Heart Rate w/o Symptoms	HR < 40	HR > 110
Weight w/ Symptoms	Daily gain > 3 Pounds	Weekly gain > 5 Pounds

These thresholds could be changed for a particular patient based on the discretion of the primary HF provider. Initially after a patient's discharge, since call study nurses are not familiar with the patient's health condition, they will call the patient to assess each trigger alert. Talking to patient over the phone, they will assess the patient's condition and decide whether they

should recommend the patient call their primary HF provider, also call the primary HF provider, initiate a 911 call, or make some other recommendations to the patient.

There are also some general and specific symptom questions that each patient will answer every day prior to performing the above-mentioned tests. These symptom questions help the nurses to evaluate the general condition as well as the heart performance of the patient, and may trigger a call to the primary HF provider if biometric thresholds are met.

Question	Trigger
Have you felt more short of breath in the last day?	Yes
Have you noticed more swelling in the last day?	Yes
Did you wake up more short of breath last night?	Yes
Did you sleep in a chair, or propped up on pillows, more than usual last night?	Yes
Have you had any light-headedness or dizziness in the last day?	Yes
Today, would you say your health is excellent, very good, good, fair, or poor?	Poor
Compared to yesterday, would you say you are feeling about the same, better, worse, or much worse?	Much worse

Like biometric parameters, the call center nurses would call the patient to assess and verify the condition. If they find the patient is symptomatic they would contact the primary HF provider to let him/her know.

Whenever the call center nurse calls the primary HF provider, she would discuss the trigger alert and could ask whether the provider tends to set a new threshold based on patient's specific conditions.

Depending on patient's symptoms and level of exacerbation, the call center nurse may call 911 and advise patient to refer to the EMS as soon as possible. In less severe conditions but still urgent, the nurse may contact patient's physician through pager or phone. In non-emergent situations, the physician may be notified through email or other means on a non-urgent basis. In sites that have installed EPIC EMR system, physicians can get notified quickly through EPIC messages system.

**Situation**	**Action**
Non-urgent condition	Non-urgent physician notification
Urgent condition	Urgent physician notification by pager/call
Emergent condition	Call 911

Since the Call Center Nurses would have access to each patient's EMR, they can look into recent hospitalization records and judge to some extent whether the readings are something that should be expected or not.

Some examples of emergent and urgent conditions in the below table:

Examples of Emergent Condition	Examples of Urgent Condition
• Irregular or rapid pulse<br>• Increased dyspnea<br>• Dyspnea while seated<br>• Extreme sweating<br>• Chest pain or chest pressure<br>• Change in mental status<br>• Blue lips, tongue, or fingernail beds<br>• Inability to speak due to dyspnea<br>• Extreme fatigue<br>• Frothy pink or copious white sputum<br>• Feeling of suffocation<br>• Sudden increase swelling in legs, feet, or abdomen<br>• Nausea and vomiting<br>• One ICD shock with symptoms or multiple ICD shocks	• Difficulty in sleeping<br>• Increased swelling in ankle<br>• Increased weakness or fatigue<br>• Frequently dry cough<br>• Increased wheezing<br>• Weight gain > 3 pounds a day<br>• Weight gain > 5 pounds a week<br>• Loss of appetite, indigestion<br>• Light headedness/dizziness<br>• Upper respiratory infection with fever and cough<br>• One time ICD shock without symptoms

Implantable Cardioverter / Defibrillator (ICD) Protocol
The following protocol should be considered If a patient notifies call center nurses about receiving shocks by his/her ICD:

A. ICD shock one time. Whenever patient reports to CCN their ICD device fired once
1) If patient is asymptomatic:
   a) CCN should instruct patient to contact ICD Clinic or HF provider as soon as possible to discuss shock event and arrange appropriate follow-up
   b) CCN should contact ICD Clinic or HF provider concerning report of ICD shock and relay information about patient's current status & instructions given to patient to call ICD clinic/provider.

2) If patient reports symptoms [i.e. chest pain, pressure, shortness of breath, abnormal heart beat, dizzy, confused or not feeling well]
   a) CCN should caution patient not to attempt to drive self to ED
   b) Based on patient's symptoms, CCN should advise patient to either have someone drive them immediately to the nearest ED or call "911"

c) CCN should contact HF provider and ICD Clinic concerning events and patient disposition

B. ICD device fired two or more times
1) CCN should advise patient to have someone drive them immediately to the nearest ED or call "911"

Making changes in biometric parameters
If primary HF provider decides to adjust his/her own desired triggers based on patient's condition, call center nurses will let Dr. Ong know by email. Changes will be effective immediately. Individualizing the parameters will minimize unnecessary alerts and trigger phone calls.

Primary HF Provider Unresponsive to Calls and Messages
In case the primary HF provider is unreachable by any means the call center nurses can ask the local site PI to help them reach the primary heart failure provider. Many physicians' contact information like pagers, backline office phone numbers, and email addresses are available through the EMR system of each site, and electronic lists have been put together for the sites where this information is not available online.

Missing Patient Readings
If a patient's readings do not show up the call center nurses will try to contact the patient up to 3 times in one day. If they can not reach a patient after making 5 phone calls at different times in 2 days, they will call the secondary contact to inquire about the patient's condition.

Readmission Notification
If a patient who is already on the trial gets hospitalized, daily readings would cease, which is a trigger for call center nurses to call patient to inquire the reason of stopping daily results. If call center nurses get notified of a patient's hospitalization, they will document it in their records. Also the primary HF provider should be informed about the patient's readmission.

Equipment Failure
In case of failure of the Ideal Life devices, patients should contact the Ideal Life toll free customer service line directly at 1-800-401-5101 for assistance. Customer service is available Monday through Friday, 6am to 5pm PST.

Subject Traveling Outside of California
In case an individual travels outside of California, he/she can carry the device and continue measurements, however the results will not be transmitted to Ideal Life server unless the patient also brings along the pod. Pods are usually not carried by patients while they are travelling. Call center nurses should avoid calling a patient if she/he is travelling abroad, and should try to identify an alternate time to call (before or after the trip). We should avoid incurring international calls if patients are traveling out of the country.

Transfer Call to EMS in Emergent Situations

In an emergent situation when the patient is alone, the call center nurse would remain on the line until the patient could talk directly with the EMS.   When CCN transfers the patient's call directly to EMS dispatcher, the CCN will immediately provide the address where the patient is physically located as well as the patient's phone number. CCN will then confirm the EMS dispatcher does not need any further information from the CCN before disconnecting from the call. CCN will immediately notify the patient's HF provider and the overall Principal Investigator (Dr. Ong).

Handling Emotional Distress and Suicide Risk
Call center nurses or research staff shall immediately notify the overall Principal Investigator (Dr. Ong) by pager and/or phone if they encounter a patient having suicidal thoughts and also contact the National Suicide Hotlines at 800-784-2433, 800-273-8255, or 800-799-4889 (Deaf Hotline). If the patient is still in the hospital, study nurses should inform the site principal investigator as well as hospital staff about patient's condition.

Patient Abuse Issues
If the call center nurses come across a case of patient abuse they should contact the primary care provider to inform him/her about the situation. It can be difficult to verify the validity of an abuse claim based on a telephone call. Based on their discretion, the nurses may also need to call adult protective services. The overall Principal Investigator (Dr. Ong) will also be notified of all such situations.

Research Related Injury, Patients Concerns or Complaints
In the event of a research related injury, or if patients have any questions, comments or concerns about the research, they are advised to immediately contact the local site principal investigator or Dr. Michael Ong at 310-794-0154, 911 Broxton Avenue, Los Angeles, CA 90024.

If patients wish to ask questions about their rights as a research participant or if they wish to voice any problems or concerns they may have about the study to someone other than the researchers, they should call the Office of the Human Research Protection Program at (310) 825-7122 or write to Office of the Human Research Protection Program, UCLA, 11000 Kinross Avenue, Suite 102, Box 951694, Los Angeles, CA 90095-1694.

Withdrawal of Participation
Patients may withdraw by their own decision at any time without consequences of any kind. The investigator may withdraw patient from participating in the research if circumstances arise which warrant doing so. If a patient is no longer able to complete a telephone survey, he/she may have to drop out, even if he/she wants to continue. The principal investigator will make the decision and let the patient know if it is not possible for him/her to continue.

Post-Discharge Surveys
All individuals (in both the control and intervention groups) will be surveyed after discharge through phone calls by research staff. The first post-discharge survey will be administered at one week, at 30 days, and at 180 days.

A hard-copy of each survey will be sent in advance to the patient's home address so that they have a chance to review the questions prior to the call. Patients may refuse to answer any questions that they do not want to answer and still remain in the study.

It is anticipated that one hour will be needed for each phone call.

Components of the first Post-Discharge (7-Day) Survey:
1) CTM
2) Hospital Questions
3) ER Questions
4) Medical Visit questions
5) Geriatric Depression Scale – Short form
6) Minnesota Living with Heart Failure Questionnaire
7) Self-Care of Heart Failure Index
8) MMAS-8 (for assessment of Medication Adherence)

Components of the second and third (last) Post-Discharge Surveys:
1) Hospital Questions
2) ER Questions
3) Medical Visit questions
4) Geriatric Depression Scale – Short form
5) Minnesota Living with Heart Failure Questionnaire
6) Self-Care of Heart Failure Index
7) MMAS-8
8) LUBBEN SOCIAL NETWORK SCALE – 6
9) MEDICAL CARE QUESTIONS
10) WHO HPQ - Absenteeism and Presenteeism Short Form

Patients' Reimbursement
Patients will receive a $10 gift card for completing each of three telephone surveys, up to a total possible of $30 for completing all three telephone surveys.
The gift card will be sent to the patient's home address by research staff after completion of each survey, and they should expect to receive the cards in 2 to 4 weeks.

Confidentiality & Patients Privacy
- The information that will be reviewed is the minimal necessary to identify potential research participants for this research.
- The information that will be obtained for identification of participants will not be reused or disclosed outside the research team, except as required by law.
- All study personnel will comply with HIPAA regulations.
- All participants will sign a HIPAA Research Authorization for Release of Personal Health Information for Research.
- To protect confidentiality of all electronic data, encryption or password protection software

and secure network servers will be used to store data in this study.

For patients providing information by paper survey, this will be done in their room during hospitalization. For patients providing information by computer-assisted telephone interview, this will be obtained from patients in their homes. The staff conducting computer-assisted telephone interviews and the centralized call center will be housed in areas where private calls can be conducted.

All patient-identifying information will be kept confidential. The survey research group will use one set of identifiers (Field IDs) in their contacts with participants. Our programmer will receive field data from the survey research group and transform the Field IDs to another set of IDs (Analytic IDs) before the data are available to the investigators. The file that links the 2 IDs will be secured and accessible only to the programmer and the principal investigator. Identifiable hard copy data will be maintained separately by the survey research group with limited access and kept only as long as needed to maintain consent files or to contact patients for follow-up surveys.

End of Trial

The total time of study for each subject is 6 months after discharge. At the end of trial intervention patients will be asked to return the devices to UCLA study center, through pre-paid shipping materials that will be sent to them. All devices upon return will be disinfected.
